# A directed weighted network-based method for drug combinations identification using drug-target and inter-target regulation

**DOI:** 10.1186/s12859-025-06321-y

**Published:** 2025-12-29

**Authors:** Shen Xiao, Yuhang Li, Jinwei Bai, Zhenhua Shen, Can Huang, Rongwu Xiang, Yuxuan Zhai, Xiwei Jiang

**Affiliations:** School of Medical Equipment, Shenyang Pharmaceutical University, Benxi, China

**Keywords:** Drug combination, Directed weighted network, Drug-target interaction, Inter-target Interaction, Regulatory effect

## Abstract

**Background:**

Drug combination is currently a promising solution in treating complex diseases due to its reducing toxicity and enhancing therapeutic efficacy. However, the accurate identification of drug combination effects remains challenging.

**Results:**

In this work, we propose a novel directed weighted network-based approach to identify drug combinations. Specifically, the network is constructed on both drug-target and inter-target interactions, together with their directed regulation. The biological processes of drug effects propagation and attenuation are modeled, aiming to capture direct and indirect drug actions on targets. By assigning weights to nodes of regulatory effects, relative distances between node sets within network can thus be computed. These distances are then analyzed to discriminate the combinatorial efficacy of various drug combinations. Empirical evaluations validate a remarkable working performance of the proposed method. Compared to existing approaches, our method is a better alternative on the task of drug combination prediction.

**Conclusion:**

The proposed method reports a creative and practical scheme for identifying drug combination effects. With the analysis of drug-target and inter-target regulatory relation, our method is more competitive in distinguishing the combinatorial efficacy, which mitigates the deficiencies of classical drug combination prediction models.

**Supplementary Information:**

The online version contains supplementary material available at 10.1186/s12859-025-06321-y.

## Introduction

To the best of our knowledge, treating or preventing diseases remains one of the greatest challenges in the field of medicine. The past decades have witnessed the domination of ‘one-target one-drug’ strategy. Despite the efficacy of targeted therapy, monotherapy fails to consider the complexity of causative factors [1].

Encouragingly, drug combination therapy gives rise to combining two or more drugs in the treatment, offering greater efficacy or lower individual drug dosages [2]. Drug combination shows its superiorities in overcoming therapy resistance, alleviating toxicity and enhancing curative effects [1, 3, 4]. As an example, the ritonavir-saquinavir combination, in human immunodeficiency virus (HIV) infection regimens, maximizes the antiviral activity of saquinavir by 50-fold-boosting its serum levels [5].

While in practice, the identification of drug combinations is generally driven by either clinical experience or serendipitous discovery, instead of established principles [6]. One classical approach for drug combination identification is a high-throughput screening experiment, which is, however, both labor-intensive and time-consuming due to the exponentially growing drug number. According to the U.S. Food and Drug Administration (FDA), the approved human drug number approximates 10^3^, indicating there are 10^5^–10^6^ pairwise combinations [7]. Motivated by the flourish of machine/deep learning algorithms, machine/deep learning-based models concentrate on predicting optimum drug combinations. Whereas, prediction outcomes of such models typically lack explainability, which does not exhibit complementary strengths, but also poses a major limitation to pharmacotherapy. For feature-based models, Zhang and Yan (SyFFM) achieve 0.75 accuracy by integrating drug-target-enzyme-anatomical therapeutic chemical features with extensive labeled data [8]. Likewise, Chen et al. (Stacked RBM) enhance feature abstraction by combining ontology fingerprints with gene expression [9]. Further, network-based approaches address system-level interactions: Network Propagation simulates drug perturbations across biological networks; Decagon-based graph convolutional network constructs heterogeneous graphs by integrating multi-category data; and Network separation identifies disease-specific clinically relevant drug combinations. [6, 10, 11]. Besides, hybrid frameworks are also widely-applied: Shi et al. (TLMCS) utilize multi-classifier fusion to reduce label bias; and Lo et al. (5NN-RF) predict clinical transition probabilities by using k-nearest-neighbor-imputed features in random forests [12, 13]. Despite their practical strengths, these methods lack insights into feature and fail to capture deep drug-target interactions.

More recently, publications report that biological networks identify their distinctiveness in biology and medicine domains. Theoretically, a biological system can be characterized as a network in a node-edge mode. Each node is a biological element of the system (e.g. protein and gene); each edge represents the interaction between two elements. Network models are crucial for shaping our understanding of complex networks, which benefits the explanation of network origin and characteristics [14]. So much is the significance of biological network that network-based methods are highlighted in clinical practices, such as drug repositioning and diseased molecular feature extraction [15, 16]. There is an ongoing trend where network-based methods are built, with a foundation on biological principles.

The drug-target interaction is one such biological system from the perspective of network biology [17]. Nonetheless, the network-based approaches in drug combination are still limited, primarily because a specified network structure is absent. Notably, the drug-target interaction performs in an ordered and directed regulatory process. The effect of drug is obtained via activating or inhibiting specific signaling pathways [18]. That is, the drugs transfer their effects to cell interior by binding to a membrane receptor at the cell surface, and further initiate the flow to downstream cells [19]. Besides, the effect attenuation within the pathway is observed during drug action [20]. The longer the signaling distance is, the more the drug effect attenuates. Current advancement is deficient in investigating the effects on network nodes and the inter-node directed regulation.

In this work, a directed weighted network-based method, according to both drug-target and target-target interaction, is proposed to identify drug combinations. During network construction, not just the type of drug action, but also the regulatory effects between nodes are exploited. In line with the drug effect propagation and attenuation while signaling, the node weight is thus determined. As far as we are aware, this work is the first that exploits inter-target regulatory relation in network establishment. In this context, the relative network distances between distinguishing node sets can be precisely derived. With the analysis of network distances, our method is capable of identifying the combinatorial efficacy of diversified drug combinations. Experiments are conducted to evaluate the prediction performance, which sets strong evidence of high accuracy. The contributions of this paper are threefold and summarized as follows:

A directed weighted network-based model is devised, which integrates both drug-target interactions and inter-target regulatory relations. Our method captures the propagation and attenuation of drug effects within biological pathways, providing a more comprehensive and accurate model for drug combination identification.

For the first time, the directed regulatory effects between targets, including activation, inhibition, expression, and repression, are explicitly modeled. Detailed analysis of drug effect propagation through pathways is performed, enhancing characterization of both direct and indirect drug-target interactions.

The computation of relative distances between node sets is leveraged to distinguish between synergistic and antagonistic drug combinations. Experiments on datasets indicate that our model is capable of identifying drug combination. The proposed method produces results considerably better than the baseline methods.

The rest of this paper is structured as follows. Section "Methodology" depicts the directed weighted network framework for drug-target relationship construction. In section "Experimental results", experimental design and method comparison are described, including data sources, network parameters, evaluation metrics, and the comparative results against seven baseline methods, followed by case studies of drug combination identification. Concluding remarks are presented in section "Conclusion".

## Methodology

**Fig. 1:**
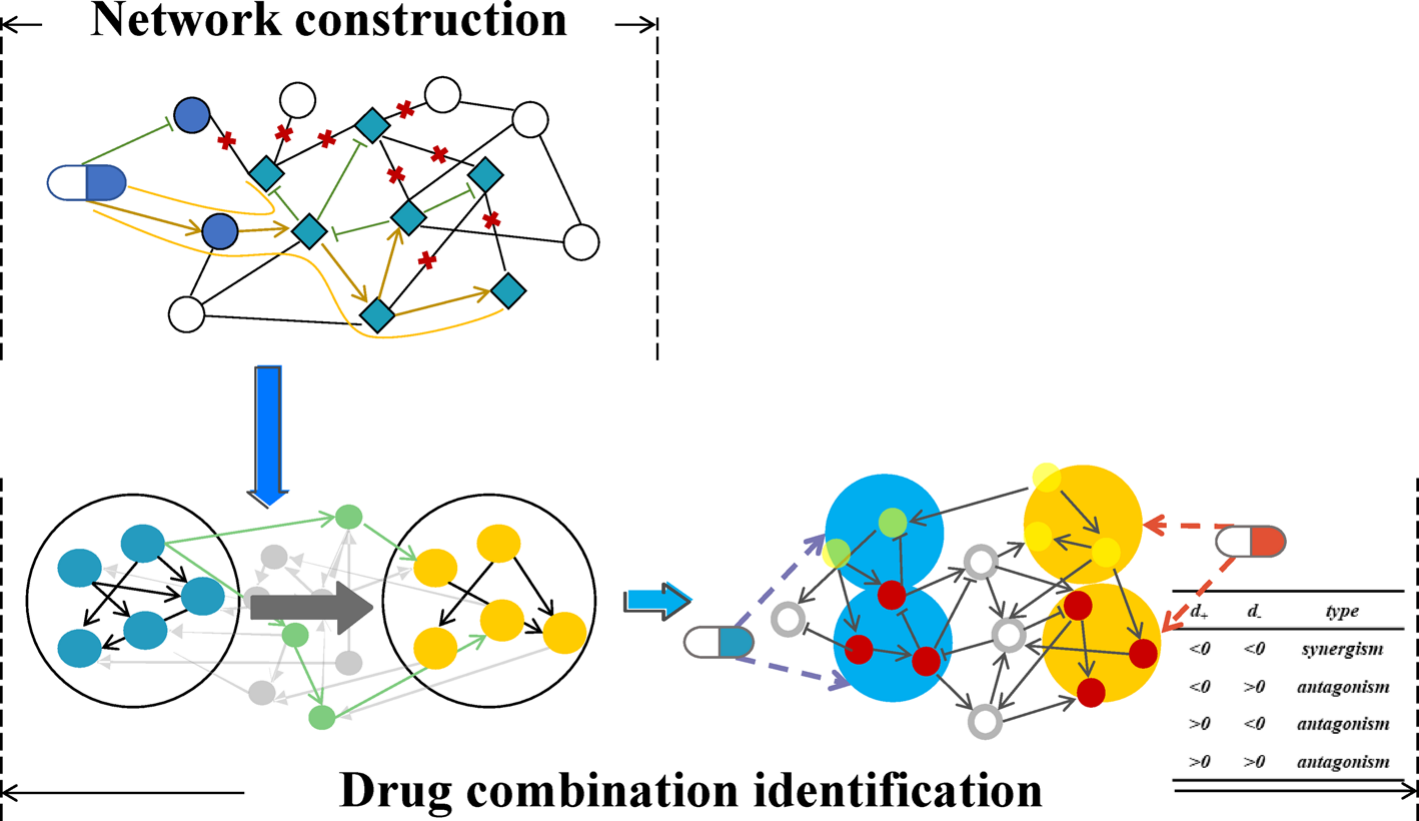
Diagram of workflow

Figure 1 presents the workflow diagram of our method. To start with, a drug-target directed weighted network is constructed, which incorporates drug-target interactions and inter-target regulation. Within the network, each target is classified as a positive- or a negative-regulated node. Subsequently, the relative distance between different categories of node sets is derived. The computation of node set distance is thus applied to determine the effect of drug combinations.

The following parts of this section describe each process in more detail.

### Network construction

**Fig. 2:**
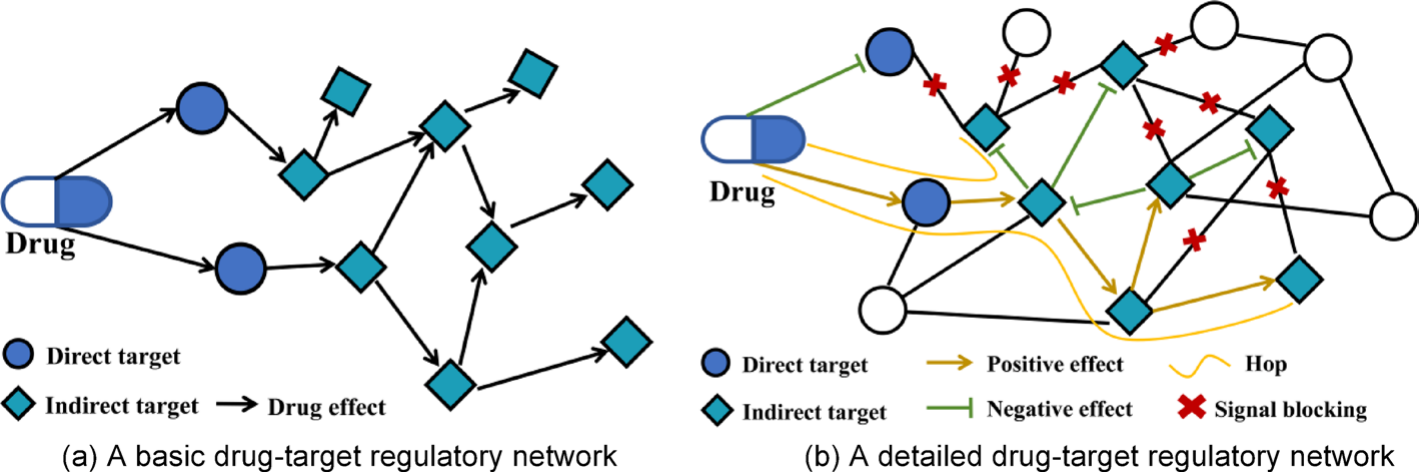
Examples of drug-target regulatory network. **a** A basic drug-target regulatory network. **b** A detailed drug-target regulatory network

As pointed out in the Introduction, the effect of a drug is performed via drug-target interaction in therapy. Following the node-edge nomenclature, a drug-target directed weighted network is constructed, with each drug-effect target as a node and the directed regulatory relation between nodes as an edge. These nodes are further divided into two categories: immediate nodes and mediate nodes. Specifically, a target that drugs directly act upon is defined as an immediate node; while a target in the downstream pathway regulated by immediate nodes is a mediate node. In line with the propagation principle, the drug effect transmits from an immediate node to mediate nodes, based on which its effect on mediate nodes can also be derived. As an example, a drug-target regulatory network is presented in Fig. 2a.

Considering the interaction among network nodes, the drug-node effect is defined as the direct regulation of drugs on their immediate nodes, while the inter-node effect indicates the downstream propagation of regulations of different nodes. Drug-node effects can be classified as activation and inhibition (based on activity regulation), and upregulation and downregulation (based on expression level regulation) [21]. Both activation and upregulation enhance the efficacy of drug-target interaction, performing positive effects on immediate nodes. In comparison, inhibition and downregulation lead to negative regulatory effects. Following such mechanism, the nodes are categorized as either positive-regulated-nodes or negative-regulated-nodes. Furthermore, the inter-node effects are defined as activation, inhibition, expression and repression. Similar to drug action, activation and expression indicate positive effects on mediate nodes while inhibition and repression represent negative effects in the context of inter-node regulation.

According to Sharma et al., a negative regulatory effect can diminish and even block downstream signaling [22]. Thereby, for a negative-regulated-node, the signaling is considered blocked at this node, making it the last mediate node in the network pathway. By contrast, the positive regulation propagates to downstream nodes along the pathway edge until no mediate node appears. In such a manner, the effect on one node can be affected by drug action from distinct edges. A more detailed description about the drug-target regulation example is given in Fig. 2b. We shall define the intensity of action (IA) as the node weight under drug action paths in the network, which is computed as:

1 $$IA=\sum \left(\pm \right)\frac{1}{{\left({\delta }_{+/-}\right)}^{hop}}$$

where $hop$ represents edge number through which drug effect transmits from the immediate node to the specific mediate node. Notably, a higher value of $hop$ causes exponential growth through overlapping downstream effects from drug-target interaction [23]. Based on biological pathway properties, $hop=2$ is adopted because it optimally balances network information capture and prevents node redundancy. The parameter $\delta$ is the attenuation coefficient of drug acting intensity, which characterizes the attenuation rate of drug effect during propagating. Specifically, ${\delta }_{+}$ and ${\delta }_{-}$ stand for the attenuation coefficients of positive regulation and negative regulation, respectively.

By calculating the value of $IA$, the nodes under drug action can be divided into two categories. If $IA<0$, the node is designated as a negative node; if $IA>0$, it is a positive node. Further, all negative nodes compose a negative node set whilst the positive nodes compose a positive node set. Specifically, the node with no drug effect ($IA=0$) is negligible during network analysis.

Note that the attenuation coefficient directly affects the signaling intensity, a differential evolution (DE) strategy is employed for $\delta$ optimization. Theoretically, DE is a heuristic method, based on a population of potential candidates, to address an optimization problem [24]. With the application of DE, an optimal $\delta$ can thus be determined. DE approach typically follows the procedures of initialization, mutation, recombination and selection [25]. More details about ED utilization are described in [26]. The DE strategy in this work is carried out as given in Algorithm 1.

**Algorithm 1.**
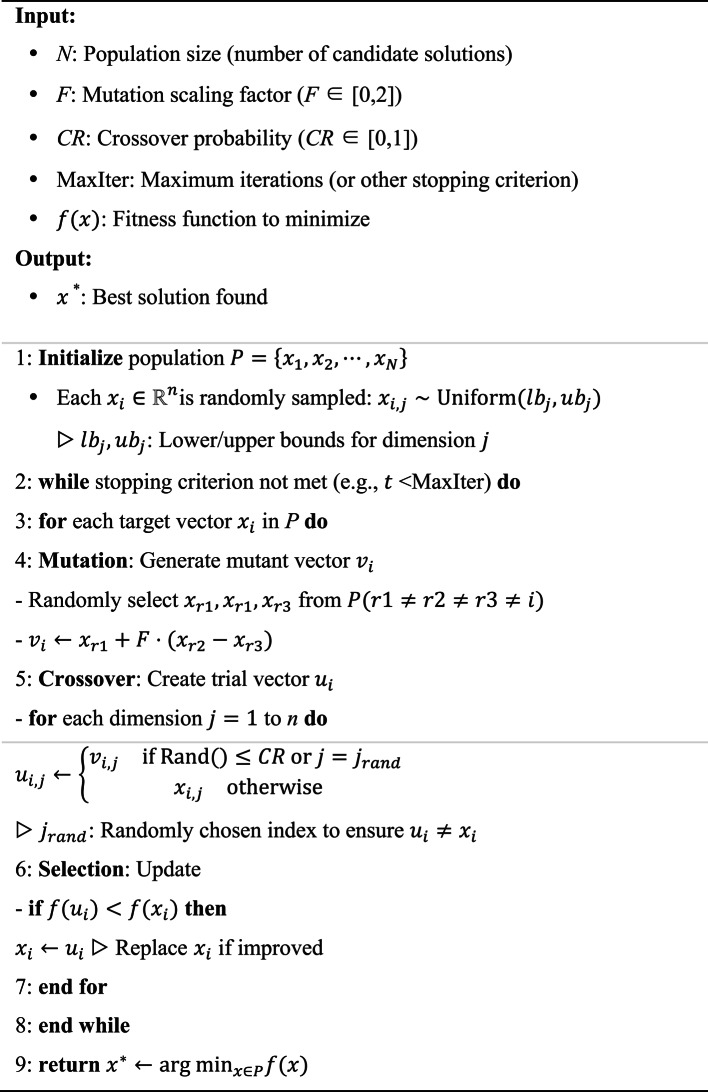
DE Algorithm

### Drug combination identification

**Fig. 3:**
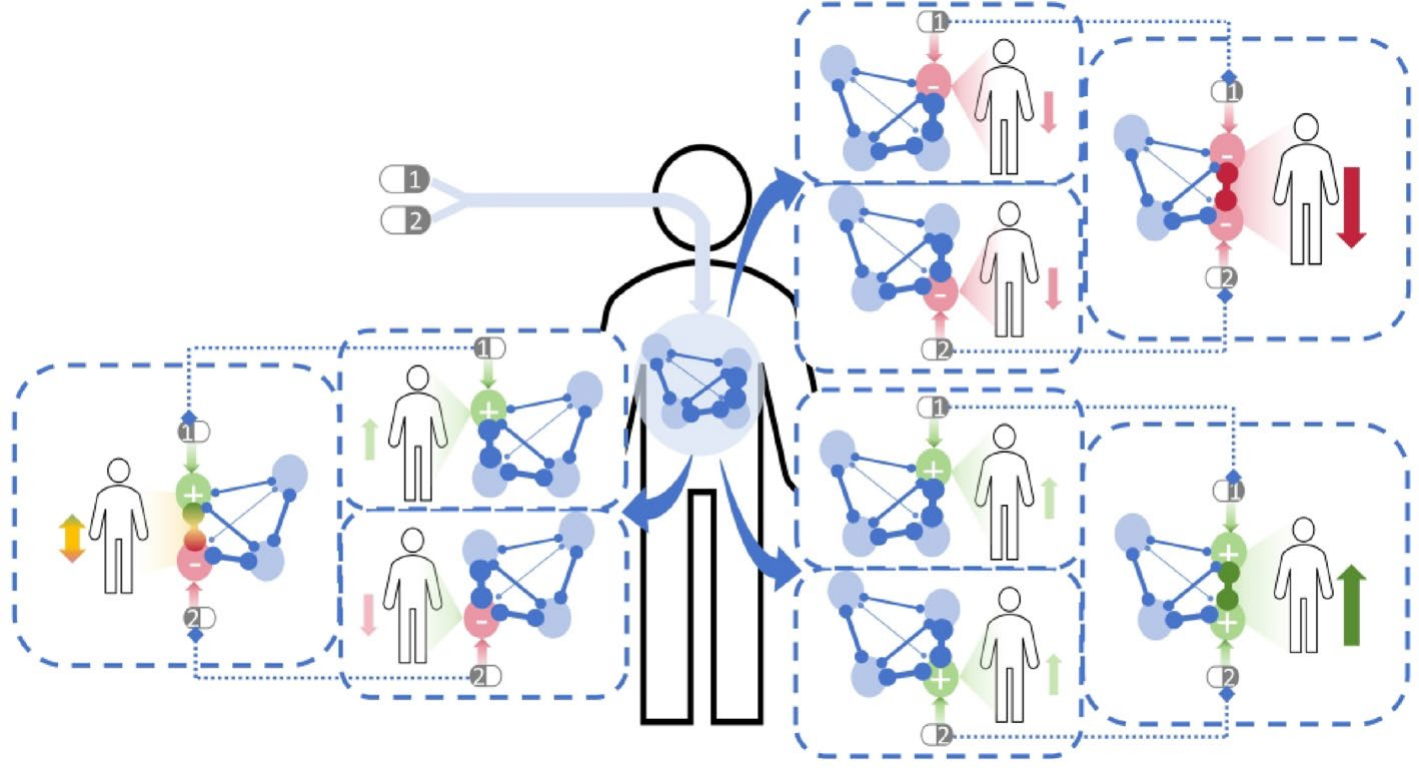
Schematic diagram of network-based drug combination interaction

Aiming at identifying the types of drug combination, a node set distance-based method is established. Within the network, the shorter the distance between two drug-effect nodes, the stronger their interaction is (Fig. 3). Following this principle, a shorter distance of two positive nodes leads to a mutual reinforcement of their positive effects; so do the negative effects of negative nodes.

**Fig. 4:**
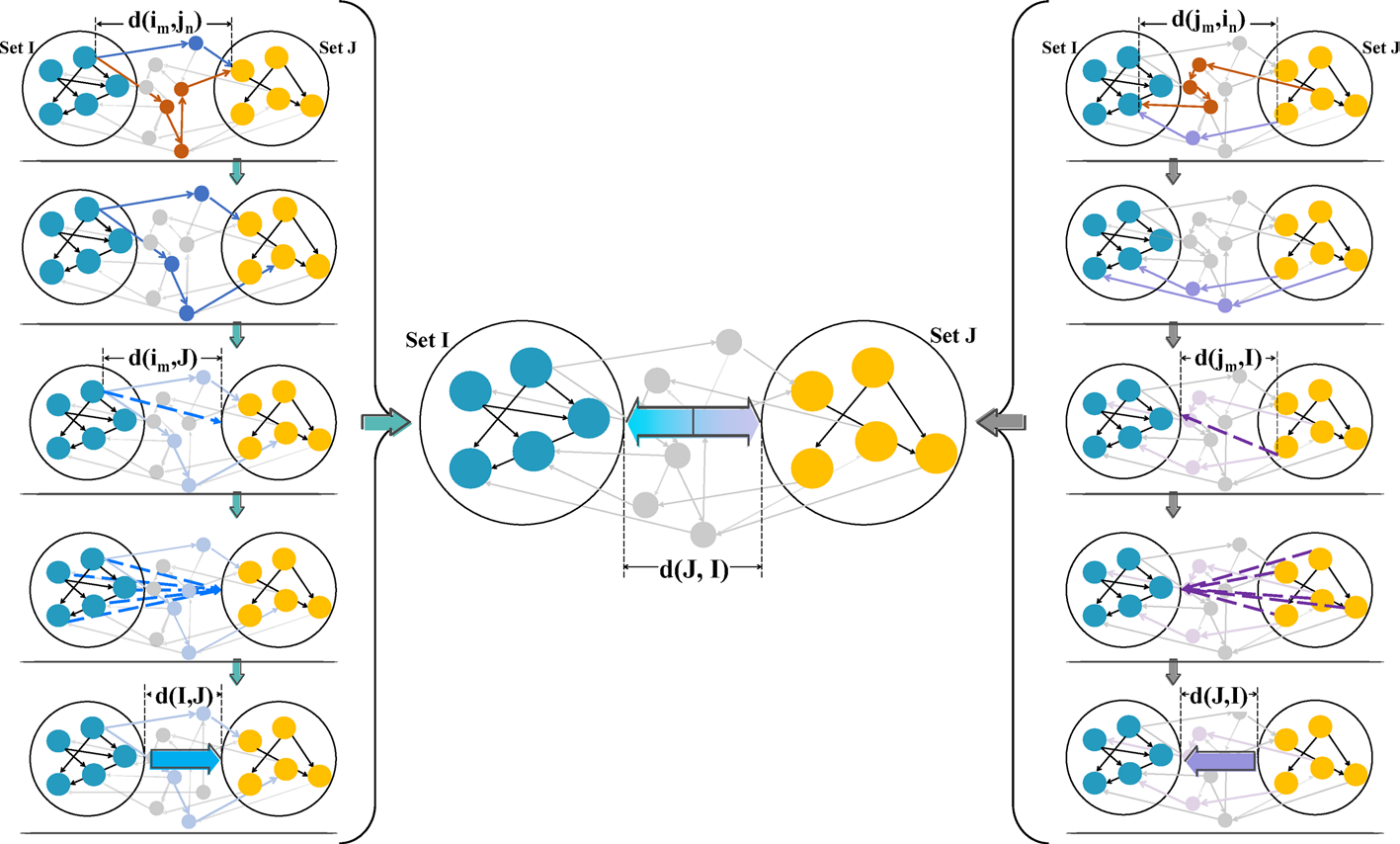
Relative network distance of node sets

For a given drug combination, let $i$ and $j$ be two nodes separately from distinguishing node sets $I$ and $J$. Their relative network distance $d\left(\left|I,J\right|\right)$ is defined as:

2 $$d\left(\left|I,J\right|\right)=\frac{1}{2}\left[\frac{1}{{n}_{I}}\sum \left(\frac{1}{{n}_{j}}\sum d\left(i,j\right)\right)+\frac{1}{{n}_{J}}\sum \left(\frac{1}{{n}_{i}}\sum d\left(j,i\right)\right)\right]$$

where $d\left(i,j\right)$ is the shortest network distance from $i$ to $j$; ${n}_{j}$ represents the number of nodes in set $J$ regulated by node $i$; ${n}_{I}$ is the number of nodes in set $I$ regulating one or more nodes in set $J$. Similarly, $d\left(j,i\right)$ refers to the shortest network distance from $j$ to $i$; ${n}_{i}$ is the number of nodes in set $I$ regulated by node $j$; ${n}_{J}$ is the number of nodes in set $J$ regulating any node in set $I$. Figure 4 illustrates the processing on relative network distance.

Based on node category, the targets for a two-drug combination can be divided into four node sets: the positive node set ${I}^{+}$ and the negative node set ${I}^{-}$ for one drug, and the positive node set ${J}^{+}$ and the negative node set ${J}^{-}$ for the other. The relative distance between the positive node sets of both drugs is defined as:

3 $${d}_{+}=d\left(\left|{I}^{+},{J}^{+}\right|\right)-min\left[d\left(\left|{I}^{+},{J}^{-}\right|\right),d\left(\left|{I}^{-},{J}^{+}\right|\right)\right]$$

Likewise, the relative distance between the negative node sets of both drugs is written as:

4 $${d}_{-}=d\left(\left|{I}^{-},{J}^{-}\right|\right)-min\left[d\left(\left|{I}^{+},{J}^{-}\right|\right),d\left(\left|{I}^{-},{J}^{+}\right|\right)\right]$$

where $d\left(\left|,\right|\right)$ stands for the relative distance between two node sets. Specifically, $d\left(\left|{I}^{+},{J}^{-}\right|\right)$ and $d\left(\left|{I}^{-},{J}^{-}\right|\right)$ are forward-regulated node sets while $d\left(\left|{I}^{+},{J}^{-}\right|\right)$ and $d\left(\left|{I}^{-},{J}^{+}\right|\right)$ are reverse-regulated node sets.

According to Eq. (3), ${d}_{+}$ indicates the difference between the distance of positive node sets and the minimum distance of reverse-regulated node sets. If ${d}_{+}<0$, the distance within positive node sets is shorter; if ${d}_{+}>0$, the minimum distance of reverse-regulated node sets is shorter. Similarly, if ${d}_{-}<0$, the distance within negative node sets is shorter; if ${d}_{-}>0$, the minimum distance of reverse-regulated node sets is shorter.

**Fig. 5:**
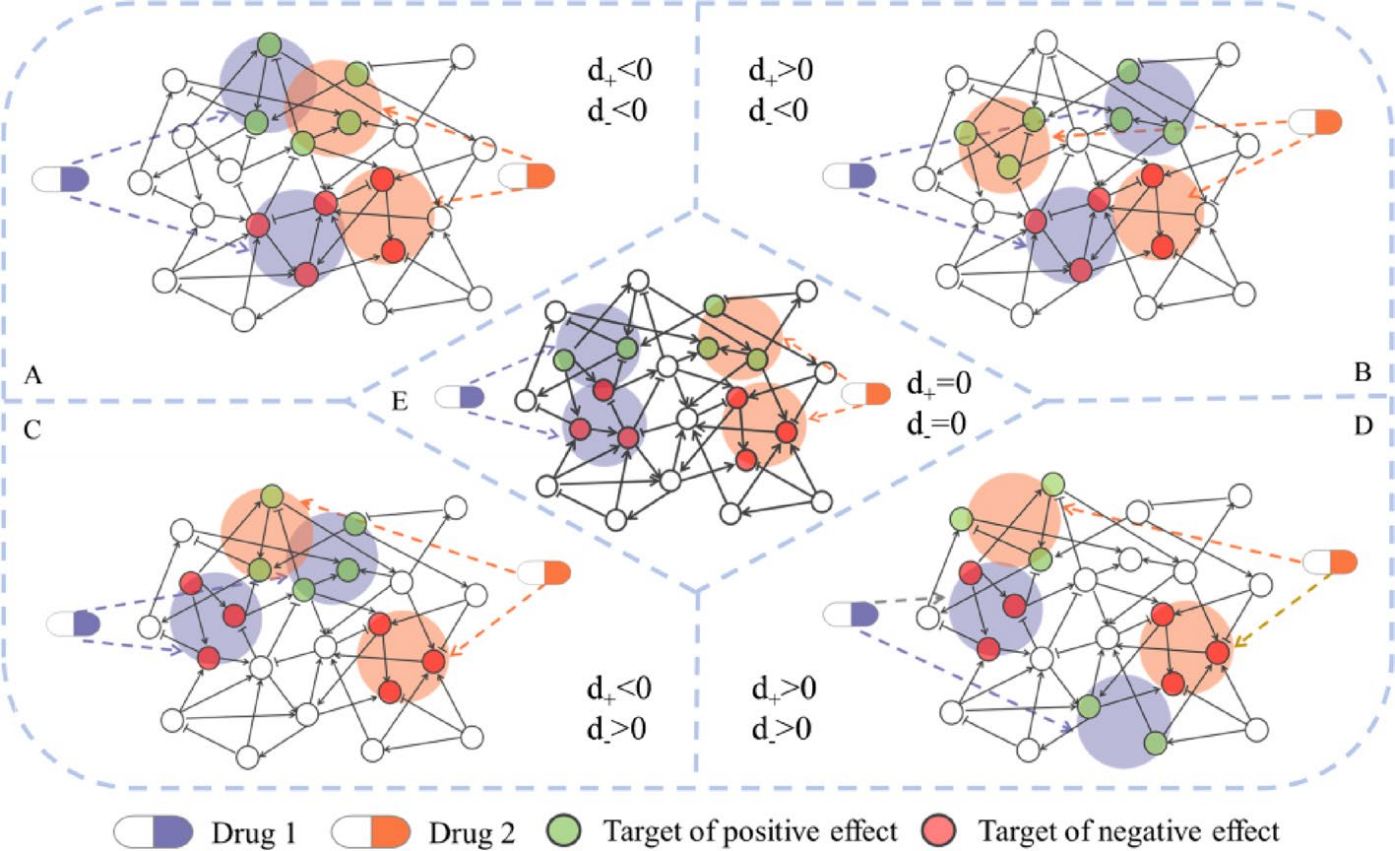
Drug combination-based network topology

With respect to a drug combination, the combinatorial efficacy can be generally categorized as synergy and antagonism. This categorization, in the context of network analysis, can be identified via relative distances of node sets. When ${d}_{+}<0$ and ${d}_{-}<0$ (Fig. 5a), the forward-regulated node sets exhibit reduced distances. Therefore, an enhanced drug effect is accessible, which leads to a synergistic drug combination. In contrast, when ${d}_{+}>0$ or ${d}_{-}>0$ (Fig. 5b–d), the distances of reverse-regulated node sets are closer. Certain effects of drugs can antagonize each other, resulting in an antagonistic drug combination. Additionally, ${d}_{+}=0$ and ${d}_{-}=0$ demonstrates the drug combination is neither synergistic nor antagonistic. As pointed out in the Network Construction Section, the $-min\left[d\left(\left|{I}^{+},{J}^{-}\right|\right),d\left(\left|{I}^{-},{J}^{+}\right|\right)\right]$ is a term that represents the signal blocking. As an example, for a drug combination, assuming the positive node set of one drug are in close proximity to the negative node set of the other drug, an increased ${d}_{+}$ value is obtained. The synergistic effect is significantly diminished, which is consistent with the pharmacological antagonism mechanism.

### Method assessment metrics

As mentioned above, the main purpose of the proposed model lies in identifying the drug combination as either synergy or antagonism. As a result, two assessment metrics are adopted to characterize the working performance of our method, which are the accuracy and the receiver operating characteristic-area under the curve (ROC-AUC).

The accuracy is computed as presented in Eq. 5:

5 $$Accuracy=\frac{TP+TN}{TP+TN+FP+FN}$$

where $TP$(True Positive), $TN$(True Negative), $FP$(False Positive) and $FN$(False Negative) separately stand for the numbers of correctly-identified synergistic combinations, correctly-identified antagonistic combinations, mis-identified antagonistic combinations and mis-identified synergistic combinations.

Moreover, the ROC-AUC is taken considering the imbalance of test set, which is computed based on Eqs. 6–8:

6 $$AUC={\int }_{0}^{1}TPR(FPR)d(FPR)$$

where

7 $$TPR=\frac{TP}{TP+FN}$$

and

8 $$FPR=\frac{FP}{FP+TN}$$

Therefore, these metrics provide a comprehensive evaluation of the model ability and reliability in predicting drug combinations.

## Experimental results

### Dataset

The drug combination dataset for working performance evaluation is sourced from the databases DrugCombDB^1^ [27] and TTD^2^ [28]. The DrugCombDB database contains multiple original drug combination datasets, such as PubMed, ASDCD and DrugBank. TTD is an online database that comprises drugs and therapeutic targets. In the updated TTD, a vast array of drug combinations from the FDA Orange Book and other datasets are provided. More statistics of the datasets are exhibited in Table 1.

**Table 1:** Statistics of datasets

Data source	Drug combination number
PubMed	441
FDA Orange Book	1363
ASDCD	548
TTD	85
DrugBank	13,397
Total count	15,834

The datasets are preprocessed as follows: Compound Identifiers (CID)-based screening and duplicates removal are implemented to obtain 13,448 drug combinations, involving 1425 drugs. Then, drug targets are extracted from the HIT^3^ database [21] via drug CID. Direct targets of 179 drugs, which relate to 204 drug combinations, are acquired. In line with the pharmacological effect observations of drug combinations documented in the DrugBank [29], 77 synergistic drug combinations and 15 antagonistic drug combinations are subjected to experiment.

In clinical practice, a drug combination acting on targets can both treat the disease indication and lead to adverse reactions. To facilitate further analysis, we define now the indication and the adverse reaction as pathological conditions. A pathological target represents the biological targets under a specific pathological condition. Considering the additive effects and mutual impacts among drug pathological conditions, we primarily focus on drug combinations relate to a single pathological condition. The screening yields 25 drug combinations, among which 21 are synergistic drug combinations and 4 are antagonistic ones. To mitigate the bias due to an imbalanced dataset, samples from the DDInter [30] database are used for antagonistic drug combinations supplementary. Thus, 17 drug combinations are randomly selected to supplement the antagonistic drug combination dataset. More details about the pathological conditions and the target number retrieved from the TTD, DisGeNet^4^ [31], and MalaCards^5^ [32] databases are given in Table 2.

**Table 2:** Statistics of pathological targets

Pathological condition	TTD	DisGeNet	MalaCards	Total count
Arrhythmia	29	559	46	608
Hypertension	58	2322	510	2420
Lymphoma	9	1548	148	1575
Tachycardia	4	73	60	128
Hypotension	15	125	20	148
Stroke	29	1159	163	1223
Diabetes	27	2803	593	2936

Furthermore, pathway enrichment on drug combination targets and their pathological targets are performed via the KOBAS database [33], which results in 326 pathway entries. Detailed information is provided in Appendix 1. Based on the pathway IDs, corresponding KGML files are derived from the KEGG database^6^ [34]. Within the KGML files, protein–protein interaction relations (PPrel) and gene expression relations (GErel) are captured, containing activation, inhibition, expression and repression. All these proteins and genes serve as both drug combination targets and pathological targets. A total of 4,282 target interaction data entries are collected. The derived targets, as well as their interactions, can thus be used in network construction.

### Examination on both types of targets

In advance of weighted directed network investigation, the drug combination targets and the corresponding pathological targets are analyzed. Targets classified as ‘successful’ and ‘elite’ are screened from the TTD and MalaCards. Both types of targets are considered highly significant candidate targets. In line with the biochemical features of protein under drug action [35], we build a protein–protein interaction (PPI) network for pathological condition targets using the STRING database [36] and compute its node parameters using CytoScape software [37]. Candidate targets of higher-than-average values in three parameters, i.e., Degree, Betweenness and Closeness, are taken as key pathological targets. Clearly, these targets are more centrally positioned in the network and exhibit stronger connection to other targets, which play a more pivotal role within the network.

At this stage, three findings are obtained from the target analysis:

1. The overlapping communities between drug combination targets and their pathological targets is scarce.
2. A drug combination target rarely serves as a pathological target.
3. Most overlapping targets are adjacent to core targets of pathological conditions, interacting with the core targets.

**Fig. 6:**
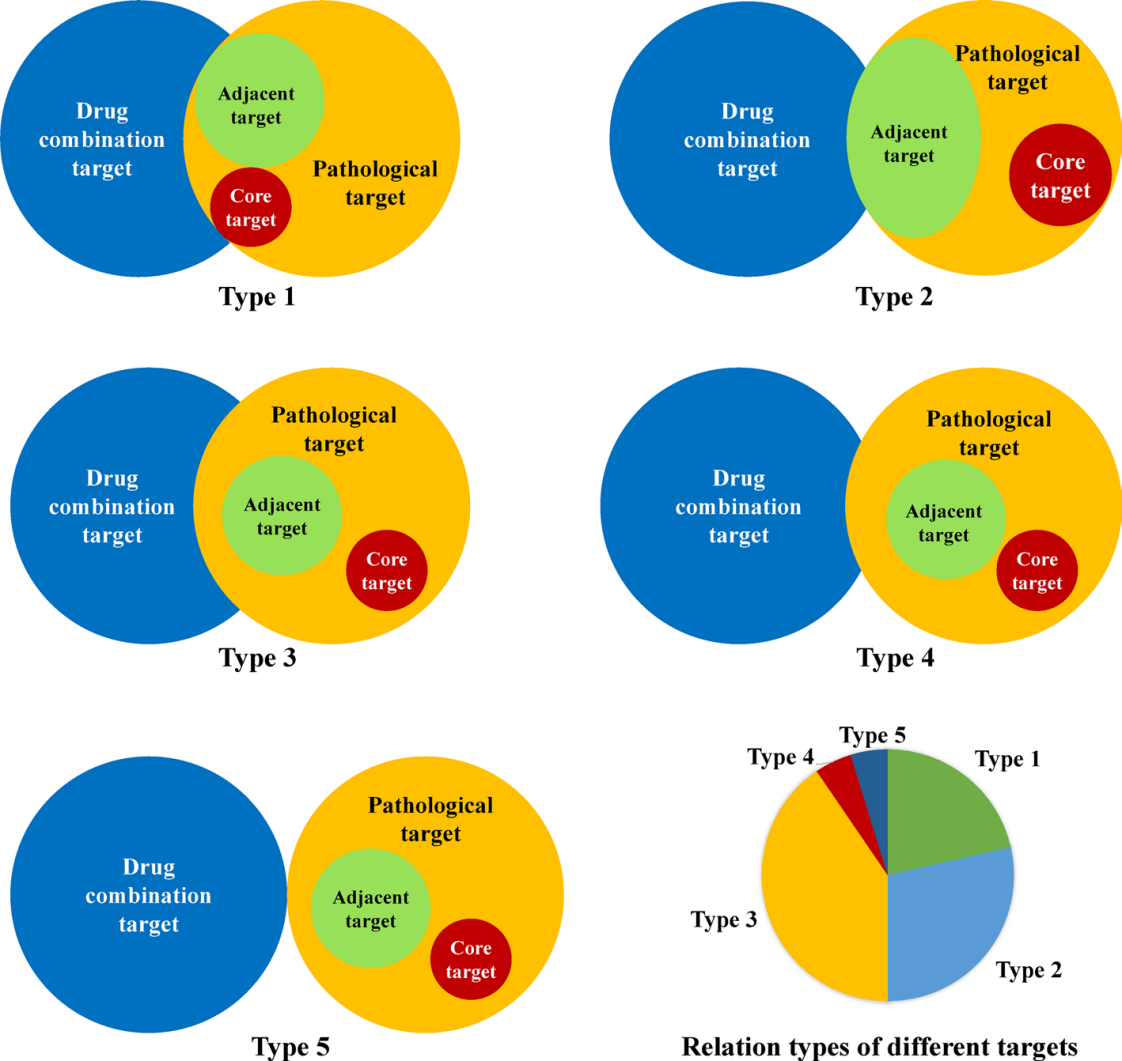
Overlap between drug combination targets and pathological targets

According to these outcomes, a drug combination not just directly acts on its targets, but also has a secondary effect on the pathological condition targets. The relations among targets are typically of five types, as given in Fig. 6. Consequently, drug combination effects on both direct and indirect targets can be further analyzed based on network establishment.

### Network configuration

**Fig. 7:**
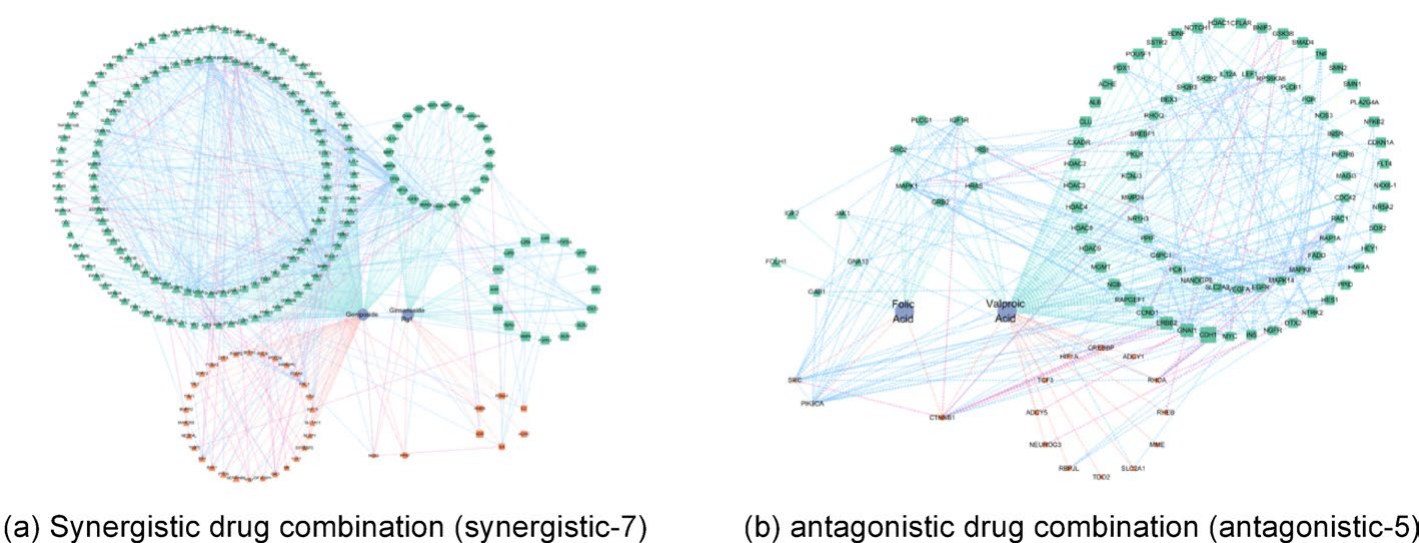
Examples of directed weighted networks on drug combination. *Green nodes: positive regulation targets; Orange nodes: negative regulation targets; Triangle nodes: targets of one drug; Square nodes: targets of the other drug; Hexagon nodes: shared targets of drug combination; Blue edges: positive regulatory effects; Pink edges: negative regulatory effects

As pointed out in section "Examination on both types of targets", the targets of drug combination and pathological condition, together with their interactions, are taken as nodes and edges on the task of network construction. Our network consists of 1840 nodes and 4282 edges, denoting 1,840 targets and 4,282 relations among targets. Full details about the configuration on each drug combination are given in Table 3. Figure 7 provides two instances of the directed weighted networks for the drug combinations. Targets are classified by either regulatory effects or drugs that act on them. Drug targets of the same category cluster and form the node set.

**Table 3:** Node and edge configuration on drug combinations

Drug combination	Node number	Edge number	Drug combination	Node number	Edge number
Synergistic-7	217	1518	Antagonistic1	269	2190
Synergistic-10	602	2998	Antagonistic-2	572	3106
Synergistic-18	614	3314	Antagonistic-3	771	3561
Synergistic-23	241	1892	Antagonistic-5	103	1260
Synergistic-24	612	3317	Antagonistic-11	476	2807
Synergistic-31	467	2914	Antagonistic-12	328	2405
Synergistic-32	623	3340	Antagonistic-13	346	2387
Synergistic-42	504	2958	Antagonistic-14	275	2029
Synergistic-43	329	2345	Antagonistic-19	243	2093
Synergistic-44	332	2378	Antagonistic-20	254	2218
Synergistic-45	642	3367	Antagonistic-26	207	1885
Synergistic-47	363	2470	Antagonistic-33	253	2034
Synergistic-48	473	2793	Antagonistic-34	141	1606
Synergistic-49	298	2148	Antagonistic-90	210	1768
Synergistic-50	629	3347			
Synergistic-65	335	2428			
Synergistic-66	427	2648			
Synergistic-67	272	2160

**Fig. 8:**
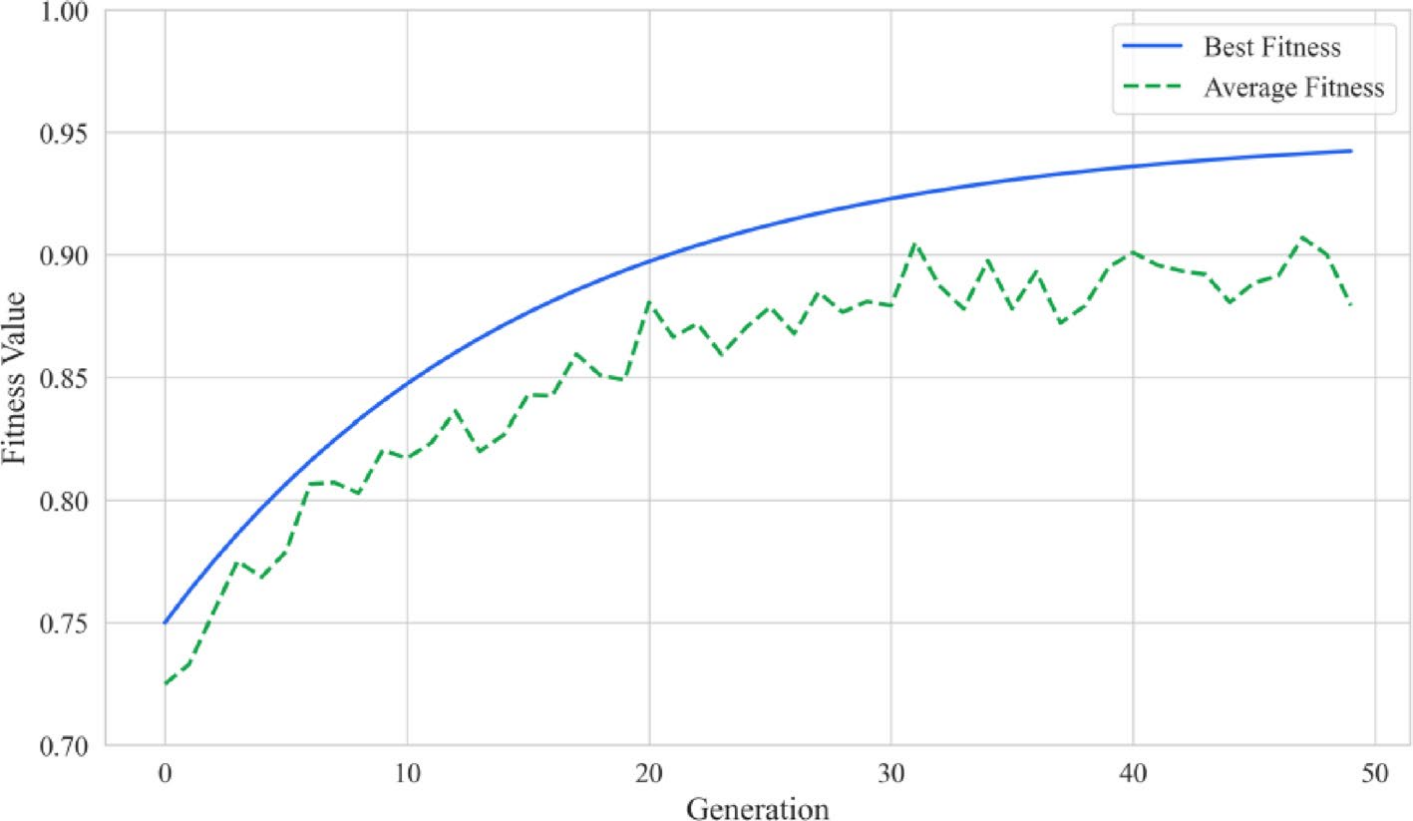
Convergence process of DE

Mediate nodes are identified on the foundation of drug-effect immediate nodes. The target weight is determined with the computation of $IA$. Optimized attenuation coefficient $\delta$ is derived via a DE strategy, with its settings in Table 4. Figure 8 depicts a stable convergence of DE, highlighting the best and average fitness values across generations. The optimal solution is reached after 40 generations.

**Table 4:** Parameter Settings of DE

Parameter	Value
Population size (NP)	50
Mutation factor (F)	0.6
Crossover rate (CR)	0.9
Maximum generations (Gmax)	100
Convergence threshold (ε)	10^–6^

### Result analysis

**Fig. 9:**
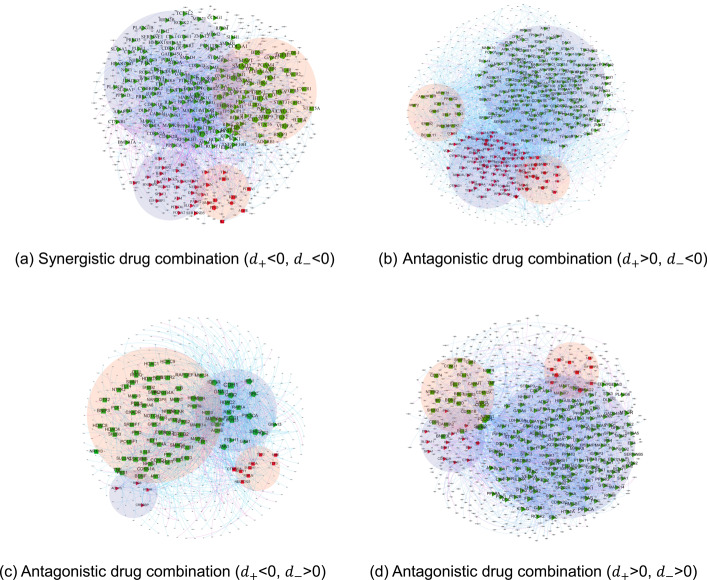
Drug combination-based network topology. *Green nodes: positive regulation targets; Red nodes: negative regulation targets; Triangle nodes: targets of one drug; Square nodes: targets of the other drug; Pentagon nodes: shared targets of drug combination; Blue edges: positive regulatory effects; Pink edges: negative regulatory effects

**Fig. 10:**
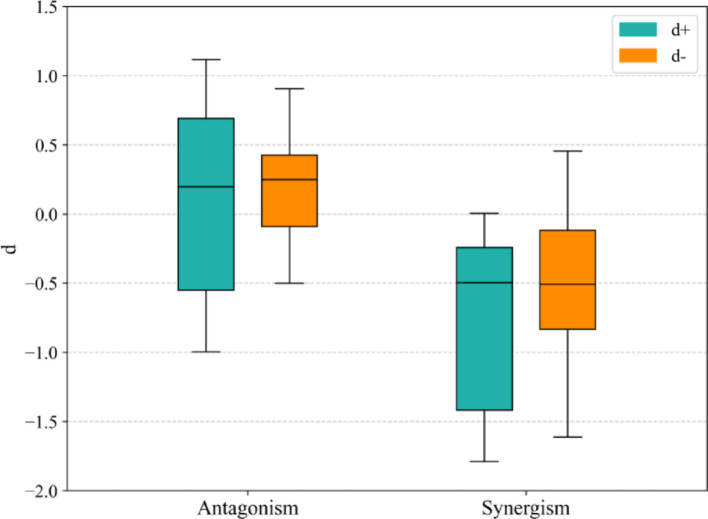
Relative distance distribution of drug combinations

**Fig. 11:**
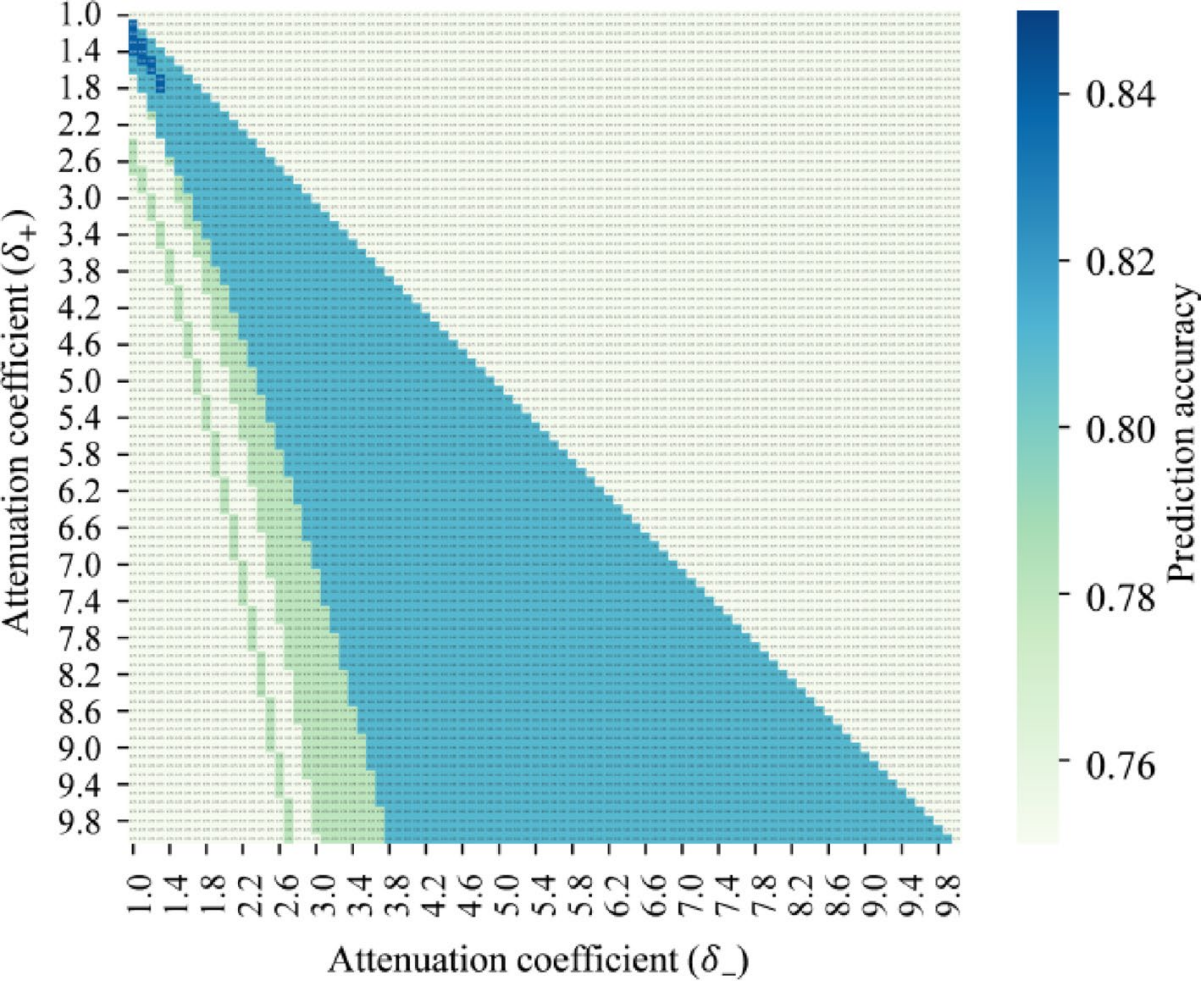
Accuracy variation with attenuation coefficients

According to section "Method assessment metrics", both the accuracy and the ROC-AUC are taken as metrics for model performance evaluation. Specifically, the proposed method achieves an accuracy of 0.84, verifying its distinctiveness in drug combination prediction. By developing the network, the topological structure associated with synergistic and antagonistic drug combinations is formed, as illustrated in Fig. 9. Similar to the network presented in Fig. 7, the computation of $IA$ value identifies both the positive and negative node sets of a drug combination. Derived from different categories of node-set-distances within the network, the relative distances of forward- and reverse-regulated node sets are precisely computed for drug combination discriminant. The distribution of relative distances is shown in Fig. 10. By employing DE, the optimal solutions of attenuation coefficients are ${\delta }_{+}=1.5$ and ${\delta }_{-}=1.1$ to reach such accuracy. A grid search is implemented to optimize the parameter values. The accuracy distribution in line with $\delta$ variation is revealed; see Fig. 11. Moreover, the accuracies on synergy and antagonism are 0.83 and 0.86, respectively, which shows negligible discrepancies. This accuracy highlights the adaptability and robustness of our method in distinguishing types of drug combinations.

Additionally, 11 drug combinations are randomly selected from the DDInter for method test. An impressive accuracy of 0.82 is attained; see Table 5. Since the distance between node sets is precisely computed, it is reasonable to expect improved reliability and thus more accurate prediction, as it is the case. From these results, one can also observe that a drug combination performs through regulatory effects among targets. Synergism is driven by actions of forward-regulated nodes while antagonism by those of reverse-regulated nodes.

**Table 5:** Method test results

Sample	$d(|I^{ + } ,J^{ + } |)$	$d(|I^{ - } ,J^{ - } |)$	$d(|I^{ + } ,J^{ - } |)$	$d(|I^{ - } ,J^{ + } |)$	$d_{ + }$	$d_{ - }$	Prediction type	Actual type
1	3.55	4.17	3.27	4.08	− 0.52	− 0.81	Synergism	Synergism
2	2.80	4.38	3.91	4.51	− 1.58	− 0.47	Synergism	Synergism
3	3.63	4.50	3.08	4.06	− 0.42	− 0.97	Synergism	Synergism
4	4.21	4.35	3.92	3.84	0.36	0.08	Antagonism	Synergism
5	2.87	4.43	3.48	4.07	− 1.20	− 0.59	Synergism	Synergism
6	2.70	4.02	3.05	3.94	− 1.24	− 0.89	Synergism	Synergism
7	3.81	3.83	3.81	3.94	− 0.01	− 0.02	Synergism	Antagonism
8	4.43	4.58	4.58	4.37	0.06	0.21	Antagonism	Antagonism
9	5.31	5.07	3.41	5.15	0.24	− 1.66	Antagonism	Antagonism
10	3.96	4.36	4.32	4.20	− 0.24	0.12	Antagonism	Antagonism
11	3.96	4.61	4.46	4.40	− 0.44	0.06	Antagonism	Antagonism

### Method comparison

To comprehensively evaluate the effectiveness of the proposed method, more benchmark approaches are taken for comparative analysis. Specifically, these baselines incorporate machine learning models, deep learning models, and network-based models. Experimental results on accuracy and receiver operating characteristic-area under the curve (ROC-AUC) are reported in Table 6.

**Table 6:** Drug combination identification results

Method	Accuracy	ROC-AUC
SyFFM [8]	0.75	–
Network Propagation [10]	–	0.75
Network separation [6]	0.70	0.59
Stacked RBM [9]	0.72	–
TLMCS [12]	–	0.82
5NN-RF [13]	–	0.81
Decagon-based GCN [11]	–	0.84
Our method	0.84	0.85

*SyFFM synergistic field-aware factorization machines, *Stacked RBM*: stacked restricted Boltzmann machine, *TLMCS* two-layer multiple classifier system, *5NN-RF* five nearest neighbors-random forests, *Decagon-based GCN* Decagon-based graph convolutional network

Clearly, the proposed method consistently outperforms all the baselines in both evaluation settings. According to Table 6, the ‘Network separation’ yields the worst results. A possible explanation is that a network without directed relation has limitations in drug-target modeling. One can easily see that the performance of SyFFM and Network Propagation is comparable. The former struggles with characterizing drug-target relation and the latter fails to classify the target types. In this context, valuable information is absent during analysis, leading to a relatively inferior result. Both TLMCS and 5NN-RF are machine learning-based classifiers that are better-performing methods than classical networks. Nonetheless, the performance enhancement of these methods depends solely on parameter tuning, due to their lacking interpretability. By contrast, Stacked RBM and Decagon-based GCN are built upon deep learning algorithms. For Stacked RBM, only genes with higher expression are retained for network construction, and the targets of indirect drug effects are neglected. Since the indirect targets involve a certain amount of interaction (section "Examination on both types of targets"), such models do not necessarily give high ROC-AUCs. The Decagon-based GCN is a state-of-the-art framework, especially in extracting information from different types of nodes and edges. Successive operations are performed on each target to integrate information from its neighbors, in order to transform the indirect drug effect among them. An ROC-AUC approaches close to that of our method is thus obtained. However, the features aggregated from neighboring nodes can generate over smoothing, which is an inherent defect of GCN. In comparison, the proposed method is established on the basis of a directed weighted network, focusing on the drug-target action and target-target interaction processes. The directed regulatory effects are leveraged in describing relative distances of node sets, which paves a way for predicting drug combination type.

## Conclusion

Drug combination does benefit the treatment of complex diseases. In this work, we propose that a network-based approach that identifies the combinatorial efficacy of drug combinations. The network is constructed upon drug-target actions, inter-target interactions, as well as their directed regulatory relations. By computing the relative distances within network, the efficacy type of drug combinations can thus be discriminated. Compared with classical network-based methods, the significance of indirect drug effect on targets is highlighted and employed for the first time. In the experiment of drug combination prediction, the proposed method achieves impressive results on distinguishing datasets. Obviously, our method is the best-performing method compared to baseline approaches on the task of drug combination identification.

The novelty of this work lies in its deep analysis of both the drug-target and inter-target regulation, based on which a method for discriminating drug combination effects is proposed. To start with, the direct and indirect actions on targets are captured to describe the directed regulatory relations, which provides a comprehension of the drug effect in a biological manner. Further, a directed weighted network based on the regulatory process is established, which models the propagation and attenuation along network pathways. Lastly, a network distance-based method is devised for distinguishing the combinatorial efficacy of drug combinations. The proposed method addresses the limitations of widely-used approaches, and offers an opportunity for the accurate identification of drug combinations.

Future work will focus on extending the analysis of drug actions and inter-target interactions to drug metabolism phases. Whether the synergistic or antagonistic effects are more prominent during metabolizing remains unsettled. While it seems clear that the proposed method can precisely identify the drug combination according to pharmacological effects, it is still an open question if it could discriminate them in all biological processes.

## Supplementary Information

Below is the link to the electronic supplementary material.

Supplementary Material 1

## Data Availability

The datasets from DrugCombDB obtained in this work are available at the following links: SynDrugComb_external[http://drugcombdb.denglab.org/download/SynDrugComb_external.xlsx], SynDrugComb_fda[http://drugcombdb.denglab.org/download/SynDrugComb_fda.xlsx], SynDrugComb_textmining[http://drugcombdb.denglab.org/download/SynDrugComb_textmining.xlsx]. The datasets from TTD obtained in this work are available at the following links: P5-01-Table 1 [https://db.idrblab.net/ttd/sites/default/files/ttd_database/P5-01-Table1.xls], P5-02-Table 2 [https://db.idrblab.net/ttd/sites/default/files/ttd_database/P5-02-Table2.xls], P5-03-Table 3 [https://db.idrblab.net/ttd/sites/default/files/ttd_database/P5-03-Table3.xls], P5-04-Table 4 [https://db.idrblab.net/ttd/sites/default/files/ttd_database/P5-04-Table4.xls]. The code is available at https://github.com/boneli/directed-weighted-network.

## Footnotes

1 http://drugcombdb.denglab.org/download.

2 https://db.idrblab.net/ttd/full-data-download.

3 http://hit2.badd-cao.net/.

4 https://www.disgenet.org/.

5 http://www.malacards.org/.

6 https://www.kegg.jp/kegg/rest/keggapi.html.
