# Supplementary material for: A directed weighted network-based method for drug combinations identification using drug-target and inter-target regulation
Source: BMC Bioinformatics. 2025 Dec 29;26:299. doi: 10.1186/s12859-025-06321-y (PMC12751882; doi:10.1186/s12859-025-06321-y)
Supplement: Supplementary file 1 — Supplementary Material 1 [file 12859_2025_6321_MOESM1_ESM.pdf]

Appendix 1 Pathway information

Number	ID	Term
1	hsa05200	Pathways in cancer
2	hsa01100	Metabolic pathways
3	hsa04151	PI3K-Akt signaling pathway
4	hsa05206	MicroRNAs in cancer
5	hsa04060	Cytokine-cytokine receptor interaction
6	hsa05163	Human cytomegalovirus infection
7	hsa04080	Neuroactive ligand-receptor interaction
8	hsa05205	Proteoglycans in cancer
9	hsa05166	Human T-cell leukemia virus 1 infection
10	hsa05161	Hepatitis B
11	hsa04010	MAPK signaling pathway
12	hsa05165	Human papillomavirus infection
13	hsa04933	AGE-RAGE signaling pathway in diabetic complications
14	hsa05418	Fluid shear stress and atherosclerosis
15	hsa04068	FoxO signaling pathway
16	hsa05167	Kaposi sarcoma-associated herpesvirus infection
17	hsa05164	Influenza A
18	hsa04932	Non-alcoholic fatty liver disease (NAFLD)
19	hsa04015	Rap1 signaling pathway
20	hsa04630	Jak-STAT signaling pathway
21	hsa05169	Epstein-Barr virus infection
22	hsa04931	Insulin resistance
23	hsa04024	cAMP signaling pathway
24	hsa05162	Measles
25	hsa04218	Cellular senescence
26	hsa05215	Prostate cancer
27	hsa04621	NOD-like receptor signaling pathway
28	hsa05152	Tuberculosis
29	hsa04910	Insulin signaling pathway
30	hsa05224	Breast cancer
31	hsa05225	Hepatocellular carcinoma
32	hsa04659	Th17 cell differentiation
33	hsa04668	TNF signaling pathway
34	hsa04066	HIF-1 signaling pathway
35	hsa05160	Hepatitis C
36	hsa04620	Toll-like receptor signaling pathway
37	hsa04062	Chemokine signaling pathway
38	hsa04714	Thermogenesis
39	hsa04210	Apoptosis

40	hsa05142	Chagas disease (American trypanosomiasis)
41	hsa04014	Ras signaling pathway
42	hsa05010	Alzheimer disease
43	hsa04152	AMPK signaling pathway
44	hsa05145	Toxoplasmosis
45	hsa04380	Osteoclast differentiation
46	hsa04020	Calcium signaling pathway
47	hsa04510	Focal adhesion
48	hsa05170	Human immunodeficiency virus 1 infection
49	hsa05135	Yersinia infection
50	hsa01522	Endocrine resistance
51	hsa05226	Gastric cancer
52	hsa05323	Rheumatoid arthritis
53	hsa04657	IL-17 signaling pathway
54	hsa05210	Colorectal cancer
55	hsa04919	Thyroid hormone signaling pathway
56	hsa04610	Complement and coagulation cascades
57	hsa01521	EGFR tyrosine kinase inhibitor resistance
58	hsa05212	Pancreatic cancer
59	hsa04371	Apelin signaling pathway
60	hsa04920	Adipocytokine signaling pathway
61	hsa04211	Longevity regulating pathway
62	hsa05321	Inflammatory bowel disease (IBD)
63	hsa04934	Cushing syndrome
64	hsa05202	Transcriptional misregulation in cancer
65	hsa04928	Parathyroid hormone synthesis, secretion and action
66	hsa04915	Estrogen signaling pathway
67	hsa04022	cGMP-PKG signaling pathway
68	hsa04722	Neurotrophin signaling pathway
69	hsa04926	Relaxin signaling pathway
70	hsa04061	Viral protein interaction with cytokine and cytokine receptor
71	hsa04658	Th1 and Th2 cell differentiation
72	hsa05144	Malaria
73	hsa04072	Phospholipase D signaling pathway
74	hsa04217	Necroptosis
75	hsa05140	Leishmaniasis
76	hsa04660	T cell receptor signaling pathway
77	hsa04625	C-type lectin receptor signaling pathway
78	hsa04064	NF-kappa B signaling pathway
79	hsa05203	Viral carcinogenesis
80	hsa04917	Prolactin signaling pathway

81	hsa04150	mTOR signaling pathway
82	hsa05235	PD-L1 expression and PD-1 checkpoint pathway in cancer
83	hsa04012	ErbB signaling pathway
84	hsa05220	Chronic myeloid leukemia
85	hsa04640	Hematopoietic cell lineage
86	hsa04979	Cholesterol metabolism
87	hsa05016	Huntington disease
88	hsa03320	PPAR signaling pathway
89	hsa05133	Pertussis
90	hsa04550	Signaling pathways regulating pluripotency of stem cells
91	hsa04930	Type II diabetes mellitus
92	hsa04071	Sphingolipid signaling pathway
93	hsa05231	Choline metabolism in cancer
94	hsa05214	Glioma
95	hsa04140	Autophagy - animal
96	hsa05223	Non-small cell lung cancer
97	hsa05221	Acute myeloid leukemia
98	hsa04921	Oxytocin signaling pathway
99	hsa04611	Platelet activation
100	hsa04520	Adherens junction
101	hsa05218	Melanoma
102	hsa04650	Natural killer cell mediated cytotoxicity
103	hsa04972	Pancreatic secretion
104	hsa04940	Type I diabetes mellitus
105	hsa05146	Amoebiasis
106	hsa04922	Glucagon signaling pathway
107	hsa04213	Longevity regulating pathway - multiple species
108	hsa05143	African trypanosomiasis
109	hsa04810	Regulation of actin cytoskeleton
110	hsa04750	Inflammatory mediator regulation of TRP channels
111	hsa05213	Endometrial cancer
112	hsa05132	Salmonella infection
113	hsa04514	Cell adhesion molecules (CAMs)
114	hsa04310	Wnt signaling pathway
115	hsa05012	Parkinson disease
116	hsa05222	Small cell lung cancer
117	hsa04350	TGF-beta signaling pathway
118	hsa01524	Platinum drug resistance
119	hsa04390	Hippo signaling pathway
120	hsa04723	Retrograde endocannabinoid signaling

121	hsa04360	Axon guidance
122	hsa04923	Regulation of lipolysis in adipocytes
123	hsa04670	Leukocyte transendothelial migration
124	hsa04916	Melanogenesis
125	hsa04728	Dopaminergic synapse
126	hsa05230	Central carbon metabolism in cancer
127	hsa05134	Legionellosis
128	hsa05416	Viral myocarditis
129	hsa04713	Circadian entrainment
130	hsa04976	Bile secretion
131	hsa04270	Vascular smooth muscle contraction
132	hsa04145	Phagosome
133	hsa05219	Bladder cancer
134	hsa04911	Insulin secretion
135	hsa04912	GnRH signaling pathway
136	hsa04950	Maturity onset diabetes of the young
137	hsa05330	Allograft rejection
138	hsa04261	Adrenergic signaling in cardiomyocytes
139	hsa05168	Herpes simplex virus 1 infection
140	hsa04612	Antigen processing and presentation
141	hsa04664	Fc epsilon RI signaling pathway
142	hsa05320	Autoimmune thyroid disease
143	hsa04672	Intestinal immune network for IgA production
144	hsa05332	Graft-versus-host disease
145	hsa05014	Amyotrophic lateral sclerosis (ALS)
146	hsa04726	Serotonergic synapse
147	hsa05211	Renal cell carcinoma
148	hsa05310	Asthma
149	hsa04975	Fat digestion and absorption
150	hsa04666	Fc gamma R-mediated phagocytosis
151	hsa04925	Aldosterone synthesis and secretion
152	hsa04137	Mitophagy - animal
153	hsa04540	Gap junction
154	hsa04918	Thyroid hormone synthesis
155	hsa04725	Cholinergic synapse
156	hsa04370	VEGF signaling pathway
157	hsa04662	B cell receptor signaling pathway
158	hsa04115	p53 signaling pathway
159	hsa04970	Salivary secretion
160	hsa05150	Staphylococcus aureus infection
161	hsa04622	RIG-I-like receptor signaling pathway
162	hsa04720	Long-term potentiation

163	hsa05131	Shigellosis
164	hsa05217	Basal cell carcinoma
165	hsa04623	Cytosolic DNA-sensing pathway
166	hsa04924	Renin secretion
167	hsa04971	Gastric acid secretion
168	hsa04960	Aldosterone-regulated sodium reabsorption
169	hsa04724	Glutamatergic synapse
170	hsa05120	Epithelial cell signaling in Helicobacter pylori infection
171	hsa04927	Cortisol synthesis and secretion
172	hsa04144	Endocytosis
173	hsa05410	Hypertrophic cardiomyopathy (HCM)
174	hsa05030	Cocaine addiction
175	hsa04110	Cell cycle
176	hsa00190	Oxidative phosphorylation
177	hsa05031	Amphetamine addiction
178	hsa01523	Antifolate resistance
179	hsa00590	Arachidonic acid metabolism
180	hsa04141	Protein processing in endoplasmic reticulum
181	hsa04530	Tight junction
182	hsa05130	Pathogenic Escherichia coli infection
183	hsa05100	Bacterial invasion of epithelial cells
184	hsa02010	ABC transporters
185	hsa05414	Dilated cardiomyopathy (DCM)
186	hsa05216	Thyroid cancer
187	hsa04914	Progesterone-mediated oocyte maturation
188	hsa04913	Ovarian steroidogenesis
189	hsa04512	ECM-receptor interaction
190	hsa00561	Glycerolipid metabolism
191	hsa01200	Carbon metabolism
192	hsa04215	Apoptosis - multiple species
193	hsa00010	Glycolysis / Gluconeogenesis
194	hsa04973	Carbohydrate digestion and absorption
195	hsa05034	Alcoholism
196	hsa04977	Vitamin digestion and absorption
197	hsa04974	Protein digestion and absorption
198	hsa04070	Phosphatidylinositol signaling system
199	hsa04710	Circadian rhythm
200	hsa00140	Steroid hormone biosynthesis
201	hsa04330	Notch signaling pathway
202	hsa00270	Cysteine and methionine metabolism
203	hsa04614	Renin-angiotensin system
204	hsa00260	Glycine, serine and threonine metabolism

205	hsa05020	Prion diseases
206	hsa00250	Alanine, aspartate and glutamate metabolism
207	hsa01230	Biosynthesis of amino acids
208	hsa05340	Primary immunodeficiency
209	hsa05032	Morphine addiction
210	hsa04961	Endocrine and other factor-regulated calcium reabsorption
211	hsa00230	Purine metabolism
212	hsa04142	Lysosome
213	hsa00982	Drug metabolism - cytochrome P450
214	hsa00591	Linoleic acid metabolism
215	hsa04730	Long-term depression
216	hsa05204	Chemical carcinogenesis
217	hsa00980	Metabolism of xenobiotics by cytochrome P450
218	hsa00480	Glutathione metabolism
219	hsa04216	Ferroptosis
220	hsa00100	Steroid biosynthesis
221	hsa00983	Drug metabolism - other enzymes
222	hsa00790	Folate biosynthesis
223	hsa00562	Inositol phosphate metabolism
224	hsa00620	Pyruvate metabolism
225	hsa04978	Mineral absorption
226	hsa05322	Systemic lupus erythematosus
227	hsa00500	Starch and sucrose metabolism
228	hsa00350	Tyrosine metabolism
229	hsa04727	GABAergic synapse
230	hsa04114	Oocyte meiosis
231	hsa05412	Arrhythmogenic right ventricular cardiomyopathy (ARVC)
232	hsa03410	Base excision repair
233	hsa00410	beta-Alanine metabolism
234	hsa04340	Hedgehog signaling pathway
235	hsa00600	Sphingolipid metabolism
236	hsa04260	Cardiac muscle contraction
237	hsa00240	Pyrimidine metabolism
238	hsa00330	Arginine and proline metabolism
239	hsa00120	Primary bile acid biosynthesis
240	hsa04964	Proximal tubule bicarbonate reclamation
241	hsa00970	Aminoacyl-tRNA biosynthesis
242	hsa00830	Retinol metabolism
243	hsa04120	Ubiquitin mediated proteolysis
244	hsa00052	Galactose metabolism

245	hsa03420	Nucleotide excision repair
246	hsa00564	Glycerophospholipid metabolism
247	hsa00051	Fructose and mannose metabolism
248	hsa00220	Arginine biosynthesis
249	hsa05110	Vibrio cholerae infection
250	hsa04962	Vasopressin-regulated water reabsorption
251	hsa04721	Synaptic vesicle cycle
252	hsa00430	Taurine and hypotaurine metabolism
253	hsa00030	Pentose phosphate pathway
254	hsa00520	Amino sugar and nucleotide sugar metabolism
255	hsa01212	Fatty acid metabolism
256	hsa00860	Porphyrin and chlorophyll metabolism
257	hsa00740	Riboflavin metabolism
258	hsa01040	Biosynthesis of unsaturated fatty acids
259	hsa03022	Basal transcription factors
260	hsa00020	Citrate cycle (TCA cycle)
261	hsa00630	Glyoxylate and dicarboxylate metabolism
262	hsa00760	Nicotinate and nicotinamide metabolism
263	hsa04146	Peroxisome
264	hsa00471	D-Glutamine and D-glutamate metabolism
265	hsa00360	Phenylalanine metabolism
266	hsa00380	Tryptophan metabolism
267	hsa00040	Pentose and glucuronate interconversions
268	hsa00440	Phosphonate and phosphinate metabolism
269	hsa00770	Pantothenate and CoA biosynthesis
270	hsa00062	Fatty acid elongation
271	hsa00071	Fatty acid degradation
272	hsa03030	DNA replication
273	hsa00565	Ether lipid metabolism
274	hsa03430	Mismatch repair
275	hsa00310	Lysine degradation
276	hsa03440	Homologous recombination
277	hsa00730	Thiamine metabolism
278	hsa00592	alpha-Linolenic acid metabolism
279	hsa00910	Nitrogen metabolism
280	hsa00640	Propanoate metabolism
281	hsa00920	Sulfur metabolism
282	hsa00061	Fatty acid biosynthesis
283	hsa00531	Glycosaminoglycan degradation
284	hsa00400	Phenylalanine, tyrosine and tryptophan biosynthesis
285	hsa05033	Nicotine addiction
286	hsa03450	Non-homologous end-joining

287	hsa00900	Terpenoid backbone biosynthesis
288	hsa03460	Fanconi anemia pathway
289	hsa00534	Glycosaminoglycan biosynthesis - heparan sulfate / heparin
290	hsa04130	SNARE interactions in vesicular transport
291	hsa04122	Sulfur relay system
292	hsa00280	Valine, leucine and isoleucine degradation
293	hsa04742	Taste transduction
294	hsa00650	Butanoate metabolism
295	hsa04744	Phototransduction
296	hsa03040	Spliceosome
297	hsa00670	One carbon pool by folate
298	hsa00130	Ubiquinone and other terpenoid-quinone biosynthesis
299	hsa04136	Autophagy - other
300	hsa03013	RNA transport
301	hsa00340	Histidine metabolism
302	hsa03060	Protein export
303	hsa00601	Glycosphingolipid biosynthesis - lacto and neolacto series
304	hsa00232	Caffeine metabolism
305	hsa00524	Neomycin, kanamycin and gentamicin biosynthesis
306	hsa03018	RNA degradation
307	hsa00450	Selenocompound metabolism
308	hsa00750	Vitamin B6 metabolism
309	hsa01210	2-Oxocarboxylic acid metabolism
310	hsa00532	Glycosaminoglycan biosynthesis - chondroitin sulfate / dermatan sulfate
311	hsa00514	Other types of O-glycan biosynthesis
312	hsa03008	Ribosome biogenesis in eukaryotes
313	hsa00510	N-Glycan biosynthesis
314	hsa00072	Synthesis and degradation of ketone bodies
315	hsa00053	Ascorbate and aldarate metabolism
316	hsa04966	Collecting duct acid secretion
317	hsa03050	Proteasome
318	hsa00512	Mucin type O-glycan biosynthesis
319	hsa00604	Glycosphingolipid biosynthesis - ganglio series
320	hsa00603	Glycosphingolipid biosynthesis - globo and isoglobo series
321	hsa00511	Other glycan degradation
322	hsa03010	Ribosome
323	hsa04392	Hippo signaling pathway - multiple species
324	hsa03015	mRNA surveillance pathway

325	hsa04740	Olfactory transduction
326	hsa00563	Glycosylphosphatidylinositol (GPI)-anchor biosynthesis
